# Knowledge and Awareness About Anabolic-Androgenic Steroid Use as a Body Shape Enhancer and Its Side Effects Among Adult Gym Participants in Jeddah, Saudi Arabia

**DOI:** 10.7759/cureus.51747

**Published:** 2024-01-06

**Authors:** Ibrahim Abumunaser, Emad Salawati, Sultan Albogami, Taher Alzahrani, Qusai Kabouha, Jamaan Alzahrani, Abdulmajeed Almalki, Nawaf Alzahrani

**Affiliations:** 1 College of Medicine, King Abdulaziz University Faculty of Medicine, Jeddah, SAU; 2 Family Medicine, King Abdulaziz University Faculty of Medicine, Jeddah, SAU

**Keywords:** adult, side effects, testosterone, anabolic androgenic steroids, aas

## Abstract

Background: Anabolic steroids, often referred to as anabolic-androgenic steroids, are steroidal androgens that include testosterone and other naturally occurring androgens, as well as synthetic androgens that are chemically linked to testosterone and have similar actions.

Material and Methods: A cross-sectional study was conducted to evaluate the knowledge and awareness about anabolic-androgenic steroid (AAS) use and its side effects among adult gym participants in Jeddah. A total of 269 adults fulfilling the inclusion criteria were included. The questionnaire covered the demographics, attitudes, and behaviors associated with AAS use and consisted of single-response questions and four multiple-response questions. All statistical methods used were two-tailed with an alpha level of 0.05, considering significance if the p-value was less than or equal to 0.05. The overall awareness score was categorized as "poor" if the students’ score was less than 60% of the overall score and "good" if the students’ score was 60% or more. Descriptive analysis was done by prescribing frequency distributions and percentages for study variables, including the adult's personal data, reasons for going to the gym, and duration.

Results: This study shows a prevalence of 6.3% of adults’ use of AAS, which was higher than in some regions in Saudi Arabia. The largest age group that uses AAS, according to this study, is 26-45 years old. A total of 185 (68.8%) were males, and a total of 185 (68.8%) were university graduates. The most reported reasons for going to the gym included fitness (63.2%), muscle building (52.8%), entertainment (39.4%), and weight loss (37.9%). 94.4% of people think that anabolic steroids are harmful to the body, and 80.3% know that misuse of anabolic steroids may lead to problems with the kidneys and liver. 75.1% of male adults and 76.8% of university graduates had good knowledge and awareness. The internet is the most common source of information.

Conclusion: Our study provides clear evidence that there is a high awareness of AAS and its side effects and a high prevalence of its use among male gym participants in Jeddah. The use of AAS is a national problem that the authorities need to act on. There is a strong need for health policy reforms to reduce the rise of AAS use among young adults.

## Introduction

Anabolic-androgenic steroids (AAS) are synthetic forms of testosterone, which is the main male sexual hormone [1]. Anabolic refers to the compound's capacity to speed up muscle growth, androgenic refers to how it magnifies male sexual features, and "steroid" refers to the compound's chemical build [2]. Testosterone is the hormone that regulates the changes in male development that take place throughout puberty and later in life, but it also plays a significant role in the anabolic and catabolic reactions of several biochemical components in tissues, such as muscles [3]. All these puberty-related changes are brought on by testosterone, but it also needs to be maintained at a particular level to preserve male sex characteristics like facial hair, a deep voice, and muscle development. Although testosterone is a hormone associated with male sex, it is also present in the female body, albeit in extremely small amounts and with no significant function [4]. Therefore, using AAS will result in all of the testosterone-related functions [5].

AASs were made for the sole purpose of treating medical conditions like steroid hormone deficiency, such as delayed puberty, in addition to diseases that cause significant loss of lean muscle mass, like cancer and AIDS patients [3]. They have also been prescribed to treat hereditary angioedema, which causes swelling of the face, arm, leg, throat, windpipe, intestines, and/or sexual organs, as well as to assist in the treatment of some types of anemia and certain types of breast cancer in some women [6,7]. Bodybuilders and athletes, both recreational and professional, have abused AASs to improve their performance, strength, and endurance [4]. AASs can be administered orally, intravenously, topically as a cream or gel, or even subcutaneously as pellets for both medicinal and non-medical purposes, with the first two routes being the most common [8]. These doses could be 10 to 100 times larger than those prescribed for therapeutic purposes. It is illegal and unsafe to use them in this way without a doctor's prescription, and doing so could shorten the user's lifespan in the long term [5].

The National Drug and Alcohol Research Center conducted research to determine which social groupings are well-known for or even predisposed to the use of AAS for purposes other than those prescribed by a physician. People who are concerned about their body images include recreational athletes, people who work in the fashion or entertainment industries, professional bodybuilders, and people who need strength for their jobs, such as bodyguards, security personnel, construction workers, police officers, and members of the armed services, as well as young men who are driven by the desire for the ideal body [9].

The degree and kind of negative effects associated with AAS vary according to the individual. These variations result from user-related characteristics, including age, sex, and body mass index (BMI), as well as drug-related factors like the type of AAS taken, route of administration, the overall duration of usage, and dose-related factors [8].

AAS can have some negative consequences, which are caused by the way they work, even when used for medical purposes and at the prescribed dose. You don't have to experience all of these or even any of them, but the typical side effects will include gynecomastia, fluid retention, dysuria, an elevated red blood cell count, low high-density lipoprotein (HDL) levels, and high low-density lipoprotein (LDL) levels, hair growth or loss depending on the area of the body, low sperm counts and infertility, and changes in libido [1].

AASs can have effects on the brain that can depress their users in a variety of ways, but most of the time, these effects are brought on by steroid abuse [1]. While there is limited research on the relationship between preexisting psychopathology and the likelihood of initiating anabolic-androgenic steroid (AAS) use, existing evidence, along with animal studies, suggests that AAS abuse or dependence can contribute to the development of distinct psychiatric disorders, heightened aggression, mood instability, abnormal eating behaviors, psychosis, and is also considered a significant risk factor for suicide [10]. Some research has substantiated the observations of an elevated incidence of psychiatric manifestations among bodybuilding athletes who use anabolic-androgenic steroids (AAS), along with an escalating frequency of AAS utilization within the female demographic [11].

The amount of AAS used for illegal purposes is typically taken without medical supervision or protocol, and the dose involved is 10 to 100 times more than that used for medicinal purposes, resulting in long-term health issues [5]. These will include issues with the kidneys, such as failure or damage, issues with the liver, such as tumors, and issues with the heart, such as hypertension and changes in blood cholesterol, all of which raise the risk of stroke and heart attack, even in young people [12]. Additionally, there will be a greater likelihood of collagen breakdown, which might result in tendon tears [13].

Male-specific side effects include baldness, gynecomastia, low sperm count and infertility, testicular atrophy, and a higher risk of prostate cancer [1]. Unusual menstrual cycles, increased body and facial hair growth, male-pattern baldness, a deeper voice, and a larger clitoris are some of the negative impacts on women [3].

The results are significant when utilized before the age of 20, when they are still regarded as teenagers since they are still growing. As a result of excessive hormone levels telling the body to prematurely end bone formation, they cause stunted growth and height [3].

In the Kingdom of Saudi Arabia, AAS use and popularity have grown significantly over time. Over the past few years, studies have been conducted in various regions of the nation to gauge AAS knowledge and the proportion of users who are aware of the threat facing us. AAS use among participants was reported in three distinct studies testing AAS knowledge in 2019: Eastern Province [2], Riyadh [4], and Jeddah [6]. These studies reported AAS use among participants with percentages of 17.69%, 29.3%, and 4.7%, respectively. All of these investigations showed that the majority of participants, including those who take AAS, had limited knowledge of the drug's negative effects [2,4,6]. In 2018, a survey was conducted to assess the attitudes and knowledge about AAS among gym users from various regions of Saudi Arabia, and the results revealed that 9.8% of them admitted to using AAS [12].

In Al-Ain district, the United Arab Emirates reported in 2008 that 7% of non-users planned to use AAS in the future [3]. In 2015, a study conducted in Kuwait revealed that while 22.7% of the participants were AAS users, only 18.2% of them had great awareness of it [14]. In Bahrain, out of the 14.6% who admitted using AAS, only 18% believed that they were bad for their health [15].

Another study conducted in Jordan among college students and athletes revealed that the major goals of taking AAS were to enhance one's physical attributes and athletic performance [5]. Only 26.3% of male gym users in Sulaymaniyah, Iraq, who participated in a study done in 2020 acknowledged using AAS actively, and 84.8% of them were aware of some of its side effects but continued using it [16].

A 2011 study in Sweden found that, out of 1752 participants, 3.9% of men reported using AAS for the first time. It also expressed a great deal of concern since we should concentrate on the risk factors that lead to the use of AAS, as the numbers are thought to be rising annually [17]. Similar research among bodybuilders in Brazil revealed that AAS usage was 20.6% [18].

## Materials and methods

A cross-sectional, self-administered online survey was carried out among Jeddah gym users from December 2022 to February 2023. The questionnaire variables cover the demographics, attitudes, and behaviors associated with AAS use, assisting the participants’ knowledge and awareness of their side effects. 

In order to demonstrate that the use of AAS is a widespread issue that requires government intervention, we performed a cross-sectional study from December 2022 to February 2023 among male and female gym users in Jeddah to assess knowledge and awareness regarding the use of AAS in bodybuilding. In addition to determining the anticipated number of AAS users and the risk factors, we also provided a brief educational video to the study participants regarding AAS and its negative effects.

To determine our sample size, "Raosoft" was employed. A minimum sample size of 385 people was taken from Jeddah's entire population, including ordinary residents who did not match the requirements for this study.

The study, was explained to all participants before they answered the questionnaire, and any male or female gym participant in Jeddah, Saudi Arabia, willing to take part was included in this study. We excluded anyone who was currently taking or had previously used AAS to treat a medical condition.

The questionnaire was adopted from a published study done in the Eastern Province of Saudi Arabia [2], is well explained in Arabic and English, and was distributed to gym participants through an online survey. All responses were anonymous. Participants’ names were not recorded, and the data remained confidential to protect privacy. Before the participant starts to fill out the questionnaire, first informed consent is obtained from all participants by explaining what this study is about, and each participant willing to take part in this study will have the option to accept, after which the questions will appear only when the participant clicks the accept button, which means that the participant has agreed to be part of this study. The 34 questions and their answers were all in Arabic and English. The questionnaire consisted of single-response questions and four multiple-response questions.

We collected personal data and asked about knowledge and awareness of AAS use. Data were collected about AAS use, the type and route of the substance, and awareness about potential complications. There were nine questions about personal data, 11 questions related to knowledge and awareness of AAS, and nine more to assess the use pattern of AAS. One question after watching the educational video about AAS side effects, which was used to test the importance of awareness of AAS side effects:

IBM Corp. Released 2012. IBM SPSS Statistics for Windows, Version 21.0. Armonk, NY: IBM Corp. was then used to process the data after it had been gathered and reviewed. All of the statistical methods utilized were two-tailed with an alpha level of 0.05 and significance determined by the p-value being less than or equal to 0.05. Regarding knowledge and awareness, each correct answer was given a 1-point score. The overall awareness level regarding anabolic steroids was assessed by summing up discrete scores for different correct awareness items. The overall awareness score was categorized as poor" if the participants’ score was less than 60% of the overall score and good" if the participants’ score was 60% or more of the overall score. Descriptive analysis was done by prescribing frequency distributions and percentages for study variables, including the adult's personal data, reasons for going to the gym, and duration. Also, knowledge and awareness items and self-reported use of anabolic steroids were tabulated, while the overall awareness level was graphed. A cross-tabulation was carried out to show the distribution of participants' overall awareness level based on their data and source of information and also to assess the relation between adults’ use of anabolic steroids and their characteristics with a Pearson chi-square test for significance and an exact probability test if there were small frequency distributions.

The Unit of Biomedical Ethics Research Committee of the Faculty of Medicine, King Abdulaziz University, approved this study (Reference No. 451-21).

## Results

A total of 269 adult gym participants out of the 403 that participated in this study and fulfilled the inclusion criteria were included. Participants' ages ranged from 18 to more than 45 years, with a mean age of 29.5 ±12.9 years. One hundred and eighty-five (68.8%) were males. A total of 185 (68.8%) were university graduates. Considering work fields, 78 (29%) were not employed or retired, 59 (21.9%) worked in the medical field, 40 (14.9%) worked in the education field, and 16 (5.9%) worked in the sports field. Monthly income less than 5000 SR was reported among 99 (36.8%), while 74 (27.5%) had a monthly income of 5000-10000 SR, and 56 (20.8%) had monthly income exceeding 15000 SR. A total of 195 (72.5%) of the participants go to the gym for more than a year. The most reported reasons for going to the gym included fitness (63.2%), muscle building (52.8%), entertainment (39.4%), and weight loss (37.9%) (Table 1).

**Table 1:** Personal characteristics of adult gym participants in Jeddah, Saudi Arabia

Personal data	Count	Column N %
Age in years		
18-25	92	34.2%
26-35	60	22.3%
36-45	58	21.6%
> 45	59	21.9%
Gender		
Male	185	68.8%
Female	84	31.2%
Marital status		
Single	136	50.6%
Married	133	49.4%
Educational level		
Secondary/below	52	19.3%
University graduate	185	68.8%
Post-graduate	32	11.9%
Work field		
Unemployed/retired	78	29.0%
Medical field	59	21.9%
Sport field	16	5.9%
Education field	40	14.9%
Private field	38	14.1%
Engineering field	22	8.2%
Military field	16	5.9%
Monthly income		
< 5000 SR	99	36.8%
5000-10000 SR	74	27.5%
10000-15000 SR	40	14.9%
> 15000 SR	56	20.8%
How long have you been going to the gym?		
< 1 year	74	27.5%
> 1 year	195	72.5%
What is your main reason for going to the gym in the first place?		
Fitness	170	63.2%
Muscle building	142	52.8%
Entertainment	106	39.4%
Weight loss	102	37.9%
Others	40	14.9%

A total of 92.9% of the study adults had heard of anabolic-androgenic steroids. The most reported sources included the internet (50.6%), friends (22.5%), and coaches (5.5%). 94.4% think that anabolic steroids are harmful to the body; 80.3% know that misuse of anabolic steroids may lead to problems with your kidneys and liver; 76.2% know of its use as a body enhancer drug in gyms; 75.1% know that misuse of anabolic steroids may cause its user to suffer from paranoia, extreme irritability, and aggression; 72.1% know that misuse of anabolic steroids may increase the risk of stroke and heart attack; and 71.4% know that misuse of anabolic steroids may cause infertility in both men and women. Only 10.8% still think of using anabolic steroids or continue using them after watching the study video (Table 2).

**Table 2:** Knowledge and awareness about anabolic-androgenic steroid use among adult gym participants in Jeddah, Saudi Arabia

Knowledge and awareness	No	%
Have you heard of anabolic-androgenic steroids?		
Yes	250	92.9%
No	19	7.1%
If yes, from where?		
Internet	128	50.6%
Friends	57	22.5%
Coach	14	5.5%
Fellow gym users	15	5.9%
Others	39	15.4%
Do you know of its use as a body enhancer drug in gyms?		
Yes	205	76.2%
No	64	23.8%
Do you think that anabolic steroids are harmful for the body?		
Yes	254	94.4%
No	15	5.6%
Do you know that the misuse of anabolic steroids may cause its user to suffer from paranoia, extreme irritability, and aggression?		
Yes	202	75.1%
No	67	24.9%
Do you know that misuse of anabolic steroids may increase risk of stroke and heart attack?		
Yes	194	72.1%
No	75	27.9%
Do you know that misuse of anabolic steroids may lead to problems to your kidneys and liver?		
Yes	216	80.3%
No	53	19.7%
Do you know that misuse of anabolic steroids may lead to infertility in both men and women?		
Yes	192	71.4%
No	77	28.6%
Do you know that misuse of anabolic steroids may lead to breast growth in men?		
Yes	181	67.3%
No	88	32.7%
Do you know that misuse of anabolic steroids may lead to menstrual irregularities in women?		
Yes	141	52.4%
No	128	47.6%
Now after watching the video, will you still think of using anabolic steroids or continue using it?		
Yes	29	10.8%
No	240	89.2%

**Figure 1:**
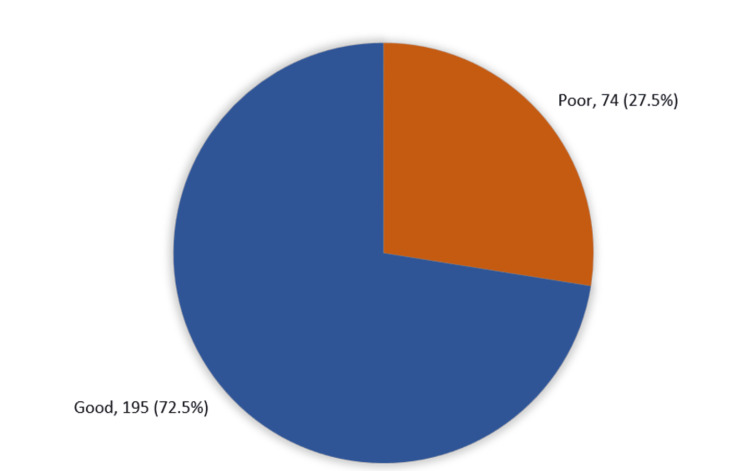
The overall study of adults’ knowledge and awareness regarding anabolic-androgenic steroid use among adult gym participants in Jeddah, Saudi Arabia

One hundred and ninety five (72.5%) of the adults had an overall good knowledge and awareness regarding androgenic steroid use, while only 74 (27.5%) had a poor knowledge level (Figure 1).

Sevnteen (6.3%) of the study adults had used anabolic steroids before, while 31 (12.3%) of non-users considered using anabolic steroids in the future. The most reported reasons for using anabolic steroids were muscle building (47.1%), participating in contests (29.4%), and other reasons (23.5%). A total of 13 (76.5%) used anabolic steroids for less than six months. As for pattern of use, five (29.4%) used anabolic steroids as oral tablets, only one used IM injections, and 11 (64.7%) used both. The most commonly used oral types were Anavar (47.1%), Proviron (47.1%), Anadrol (41.2%), Winstrol (41.2%), Dianabol (23.5%), and Turinabol (17.6%). The most commonly used injections included Deca-Durabolin (52.9%), Sustanon (41.2%), Primobolan (35.5%), and Depot (35.5%). A total of five users (29.4%) got these anabolic steroids from the internet, and three (17.6%) got them from friends. As for the effects noticed, 12 (70.6%) reported an increase in performance, six (35.3%) reported an increase in muscle mass, and six (35.3%) reported an increase in the rate of fat burn, while eight (47.1%) reported on all of them (Table 3).

**Table 3:** Adult gym participants use patterns of anabolic-androgenic steroids, Jeddah, Saudi Arabia

Practice items		No	%
Have you ever used anabolic steroids before?	Yes	17	6.3%
	No	252	93.7%
If your answer in question 13 was "no", will you consider using anabolic steroids in the future? (n=252)	Yes	31	12.3%
	No	221	87.7%
If you use or have used anabolic steroids, what was the reason? (n=17)	For muscle building	8	47.1%
	To participate in contests	5	29.4%
	Other reasons	4	23.5%
How long have you used it? (n=17)	Less than 6 months	13	76.5%
	6 months to year	3	17.6%
	More than a year	1	5.9%
How do you take it? (n=17)	Oral tablets	5	29.4%
	IM injections	1	5.9%
	Both of them	11	64.7%
If oral tablets, which of these do you use? (n=17)	Anavar	8	47.1%
	Proviron	8	47.1%
	Anadrol	7	41.2%
	Winstrol	7	41.2%
	Dianabol	4	23.5%
	Turinabol	3	17.6%
If intramuscular injection, which of the following do you use? (n=17)	Deca-durabolin	9	52.9%
	Sustanon	7	41.2%
	Primobolan	6	35.3%
	Depot	6	35.3%
	Others	10	58.8%
From where or whom do you get these anabolic steroids? (n=17)	Internet	5	29.4%
	Friends	3	17.6%
	Coach	1	5.9%
	Others	8	47.1%
When using anabolic steroids have you noticed any of its positive effects while going to the gym? (n=17)	Increase in performance	12	70.6%
	Increase in muscle mass	6	35.3%
	Increase rate of fat burn	6	35.3%
	All of the above	8	47.1%
	None of the above	1	5.9%

75.1% of male adults had good knowledge and awareness regarding anabolic steroids versus 66.7% of female adults, with recorded statistical significance (P =.049). Also, 76.8% of university graduates had good overall knowledge and awareness in comparison to 56.3% of those with postgraduate degrees (P =.036). Good knowledge was detected among 78.5% of adults who used anabolic steroids for more than a year, compared to 56.8% of others who used them for less than a year (P =.001) (Table 4).

**Table 4:** Factors associated with the study of adults’ knowledge and awareness regarding anabolic-androgenic steroids P: Pearson X2 test, $: Exact probability test, * P < 0.05 (significant)

Factors	Knowledge & awareness level					p-value
	Poor	Good				
	No	%	No	%		
Age in years						.770
18-25	27	29.3%	65	70.7%		
26-35	15	25.0%	45	75.0%		
36-45	18	31.0%	40	69.0%		
> 45	14	23.7%	45	76.3%		
Gender						.049*
Male	46	24.9%	139	75.1%		
Female	28	33.3%	56	66.7%		
Marital status						.210
Single	42	30.9%	94	69.1%		
Married	32	24.1%	101	75.9%		
Educational level						.036*
Secondary/below	17	32.7%	35	67.3%		
University graduate	43	23.2%	142	76.8%		
Post-graduate	14	43.8%	18	56.3%		
Work field						.086
Unemployed/retired	30	38.5%	48	61.5%		
Medical field	13	22.0%	46	78.0%		
Sport field	3	18.8%	13	81.3%		
Education field	13	32.5%	27	67.5%		
Private field	10	26.3%	28	73.7%		
Engineering field	2	9.1%	20	90.9%		
Military field	3	18.8%	13	81.3%		
Monthly income						.571
< 5000 SR	31	31.3%	68	68.7%		
5000-10000 SR	19	25.7%	55	74.3%		
10000-15000 SR	8	20.0%	32	80.0%		
> 15000 SR	16	28.6%	40	71.4%		
Source of information						.511^$^
Internet	33	25.8%	95	74.2%		
Friends	17	29.8%	40	70.2%		
Coach	3	21.4%	11	78.6%		
Fellow gym users	5	33.3%	10	66.7%		
Others	6	15.4%	33	84.6%		
How long have you been going to the gym?						.001*
< 1 year	32	43.2%	42	56.8%		
> 1 year	42	21.5%	153	78.5%		
Are you still using OR have used anabolic steroids?						.133^$^
Yes	2	11.8%	15	88.2%		
No	72	28.6%	180	71.4%		

A total of 8.6% of male adults used AAS versus 1.2% of females (P =.020). Also, 25% of those who work in military fields used AAS compared to none of the unemployed (P =.003). Likewise, 9.9% of those who go to the gym for muscle building used AAS versus 1% of those who went for weight loss (P =.005). ASS was used among 28.6% of those who had information from their coach in comparison to 3.5% of those who had information from friends (P =.002) (Table 5).

**Table 5:** Factors associated with adults’ use of anabolic-androgenic steroids P: Pearson X2 test, $: Exact probability test, * P < 0.05 (significant)

Factors	Have you ever used anabolic steroids before?					p-value
	Yes			No		
	No		%	No	%	
Age in years						.127^$^
18-25	4	4.3%		88	95.7%	
26-35	6	10.0%		54	90.0%	
36-45	6	10.3%		52	89.7%	
> 45	1	1.7%		58	98.3%	
Gender						.020*
Male	16	8.6%		169	91.4%	
Female	1	1.2%		83	98.8%	
Marital status						.424
Single	7	5.1%		129	94.9%	
Married	10	7.5%		123	92.5%	
Educational level						.217^$^
Secondary/below	6	11.5%		46	88.5%	
University graduate	9	4.9%		176	95.1%	
Post-graduate	2	6.3%		30	93.8%	
Work field						.003*^$^
Unemployed/retired	0	0.0%		78	100.0%	
Medical field	2	3.4%		57	96.6%	
Sport field	2	12.5%		14	87.5%	
Education field	2	5.0%		38	95.0%	
Private field	4	10.5%		34	89.5%	
Engineering field	3	13.6%		19	86.4%	
Military field	4	25.0%		12	75.0%	
Monthly income						.516^$^
< 5000 SR	4	4.0%		95	96.0%	
5000-10000 SR	7	9.5%		67	90.5%	
10000-15000 SR	3	7.5%		37	92.5%	
> 15000 SR	3	5.4%		53	94.6%	
What is your main reason for going to the gym in the first place?						.005*
Muscle building	14	9.9%		128	90.1%	
Fitness	9	5.3%		161	94.7%	
Weight loss	1	1.0%		101	99.0%	
Entertainment	9	8.5%		97	91.5%	
Others	2	5.0%		38	95.0%	
If yes, from where?						.002*^$^
Internet	5	3.9%		123	96.1%	
Friends	2	3.5%		55	96.5%	
Coach	4	28.6%		10	71.4%	
Fellow gym users	3	20.0%		12	80.0%	
Others	3	7.7%		36	92.3%	

## Discussion

Our study aimed to assess knowledge and awareness about anabolic-androgenic drugs and their side effects among adult gym participants in Jeddah, Saudi Arabia. This study found that the most popular resource for learning about anabolic androgenic drugs and their side effects is the internet (50.6%). Moreover, our findings imply that 94% of the gym participants in Jeddah are quite knowledgeable about anabolic androgenic drugs and their adverse effects.

Consequently, this study shows a 6.3% prevalence of adults’ use of AAS in our study in Jeddah, which is lower than comparable studies conducted in Saudi Arabia.

AAS use was prevalent in Riyadh at 29.3 [4], the Eastern Province at 17.69% [2], and the Jazan region at 31% [1]. Our study's prevalence, however, was higher than that of several Saudi Arabian provinces, such as the North and South regions, where AAS use rates were, respectively, 5.7% and 2.3% [10]. Furthermore, our study's findings indicate that people between the ages of 26 and 45 are the ones most likely to use AAS, which is in line with those of other studies. In the Jazan region, the Eastern Province, and Riyadh, the majority of AAS users were older than 25 [1,2,4].

According to our study’s result, the most common sources of AAS purchases were through the internet, friends, coaches, or others, with percentages of 29.4%, 17.6%, 5.9%, and 47.1%, respectively. In comparison with similar studies, the most common source of purchase in the Eastern province was coaches, with 55.06% [2]. The main reason for AAS use was for the purpose of muscle building, which was shown in both our study and the one done in the Eastern province with the following percentages of 47.1% and 68.54% [2], respectively. When discussing the most popular type of AAS used in our study, the one in the Eastern province and the study in Jazan, they showed the following percentages in this order: the most used type, with 58.8%, was the choice others, which was more than any type mentioned in our questionnaire, while Anavar with 47.1% and deca-durabolin with 52.9%. In the study done in the Eastern province, the most used was Anavar, with 61.8% [2], and in Jazan, deca-durabolin, with 57.6% [1].

One of the most proven positive effects of AAS was increased performance, which was reported by 70.6% of AAS users in this study, followed by increased muscle mass, reported by only 35.3%.

Females were involved in our study for the first time in Saudi Arabia. As a result, female participants made up 31.2% of the total participants. In addition, 1.2% of total female participants used AAS out of the total AAS users. For the first time in Saudi Arabia, female participants were included in our study. Women made up 31.2% of the total participants as a result. Additionally, out of all female participants, 1.2% used AAS.

The questionnaire included an educational video about the function and side effects of AAS, which was not the case in the majority of comparable trials. Our findings indicate that 12.3% of individuals who are not now using or have never used anabolic steroids thought about using them in the future; however, after watching the educational video, only 10.8% of participants still considered the thought. This demonstrates the significance and value of the educational video as it affected the decisions made by the participants, highlighting the need to provide education from reputable sources to everyone, not just gym members.

Finally, the significant limitation faced in this study is its prevalence and accuracy, which largely rest on the honesty and transparency of the participants. Because of underreporting, it is thought that the actual number of AAS users is substantially higher than what is displayed.

## Conclusions

In conclusion, the results of this study provide clear evidence that there is high awareness about AAS and its side effects among adult gym participants of both genders in Jeddah. However, the lifetime prevalence of AAS use among adult gym participants in Jeddah is probably higher than reported in this study. Our research indicates that the primary source of AAS was the internet.

Finally, because the issue cannot be resolved by educating gym users alone, our study can help persuade local authorities to launch a nationwide effort to inform the general public as well as gym users about AAS in general and its side effects.
